# Does different subfertility etiology affect pregnancy rates in intrauterine insemination cycles?

**DOI:** 10.3906/sag-1902-200

**Published:** 2019-10-24

**Authors:** Batuhan TURGAY*, Yavuz Emre ŞÜKÜR, Batuhan ÖZMEN, Ruşen AYTAÇ, Cem Somer ATABEKOĞLU, Bülent BERKER, Murat SÖNMEZER

**Affiliations:** 1 Department of Obstetrics and Gynecology, School of Medicine, Ankara University Turkey

**Keywords:** Intrauterine insemination, assisted reproductive technology, fertility etiology

## Abstract

**Background/aim:**

To investigate the relationship between subfertility etiologies and success rates in controlled ovarian stimulation and intrauterine insemination (COS–IUI) cycles.

**Materials and methods:**

The medical records of 218 couples who applied to a university-based fertility center were analyzed retrospectively. Detailed infertility examination data and pregnancy outcomes were compared according to different subfertility etiologies. The study groups with regard to subfertility etiologies were minimal–mild endometriosis, unexplained infertility, and mild male infertility. The primary outcome measure was live birth rate.

**Results:**

There were no statistically significant differences between the groups regarding demographics except for total motile sperm count. Live birth rates in the male infertility group were comparable to the endometriosis and unexpected infertility groups (6.6%, 11.9%, and 10.3%, respectively; P = 0.63).

**Conclusion:**

The success rate of the mild male subfertility group following COS–IUI cycles for live birth rates was similar to those of the endometriosis and unexplained subfertility groups.

## 1. Introduction 

Subfertility is defined as the failure to conceive after 1 year of regular, unprotected intercourse. It affects approximately 8%–15% of couples [1]. Intrauterine insemination (IUI) is a procedure in which processed and concentrated motile sperm are placed directly into the uterine cavity with an insemination canula. Intrauterine insemination, with or without ovarian stimulation, is frequently used as a first line infertility treatment because it is a relatively inexpensive, less invasive, and effective method which is indicated for different subfertility etiologies [2–4]. Mild male subfertility, minimal–mild endometriosis, unexplained subfertility, and several physical–psychosexual problems are major indications for IUI [5] National Institute for Health and Clinical Excellence (NICE) guidelines. 2013 Feb. https://www.nice.org.uk/guidance/cg156/ifp/chapter/intrauterine-insemination.. 

The intrauterine insemination procedure can be applied with normal menstrual cycles or controlled ovarian stimulation (COS). Clomiphene citrate, letrozole, or gonadotropins can be used for COS. Furthermore, it is reported that the best pregnancy rate is achieved by COS–IUI using gonadotropins when compared to other treatments [6–8]. 

The live birth rate with the IUI procedure has been reported as between 8.5% and 12.2% in different studies [9]. There is sufficient evidence that COS–IUI improves pregnancy rates in unexplained subfertility and miminal–mild endometriosis, but its value for mild male factor subfertility is still debated [4,10–14]. The aim of the present study was to compare live birth rates after COS–IUI in subfertile patients with male infertility, minimal–mild endometriosis, and unexplained infertility. 

## 2. Materials and methods

Data of all infertile couples who underwent COS–IUI in a university-based fertility center between 2015 and 2016 were collected retrospectively from medical records. The first cycles of each couple in our unit were analyzed to prevent crossover bias. The present study was approved by the Institutional Review Board (approval no: 050.01.04-E.8946). The inclusion criteria were age between 18 and 35 years and a treatment plan of IUI. The exclusion criteria were stage 3 or 4 endometriosis, decreased ovarian reserve (a serum FSH level greater than 12 IU/L or a baseline follicle count 8 or less) [15,16], recurrent pregnancy loss (two or more miscarriages), and advanced male factor infertility (total motile sperm count less than 5 × 106) [17]. All couples underwent standard infertility evaluation prior to IUI including hysterosalpingography, semen analysis, baseline serum hormonal assays, midluteal progesterone levels, and transvaginal ultrasonography. Semen analysis was done according to World Health Organization criteria (2010). 

 Three groups of patients were included in this study: minimal–mild endometriosis, unexplained infertility, and mild male factor infertility. All of the endometriosis patients had already been diagnosed by laparoscopic procedure indicated according to basal infertility examination; minimal–mild endometriosis patients according to American Fertility Society scoring were included in this study. During laparoscopy, endometriotic nodules were cauterized or excised and pelvic adhesions were lysed to achieve normal pelvic anatomy. In addition, chromopertubation was performed to assess tubal patency. Controlled ovarian stimulation and an IUI procedure were planned for the earliest following surgery. None of the patients in the endometriosis group were administered any adjuvant hormonal therapy before the COS–IUI cycle. The mild male subfertility group was defined by semen samples with a total motile sperm count (TMSC) <20 × 106/mL, normal morphology <30%, or progressive motility (grade A + B) <40% before sperm preparation. 

Ovulation induction was achieved with gonadotropin injection. Injections were administered daily, starting on day 2 or 3 of the menstrual cycle. The dose was adjusted according to ultrasonographic findings. Ovulation was triggered by recombinant hCG when at least one follicle was greater than 18 mm in mean diameter. Single IUI was performed with a disposable catheter 36 hours after ovulation was triggered. Semen was collected in sterile containers by masturbation after 2–4 days’ refrain from ejaculation. The continuous density gradient centrifugation technique was performed for semen processing by a single technician. The luteal phase was supported by vaginal progesterone preparations daily (200 mg 1 × 1 daily). Two weeks after insemination, plasma β-hCG levels were measured. The primary outcome measure was live birth rate. 

Data analyses were performed by using SPSS Version 21.0 (IBM Corporation, Armonk, NYC, USA). A Shapiro–Wilk test was used to test distribution of normality. According to the results, parametric tests were preferred. Continuous variables were compared with a one-way ANOVA test. Categorical variables were compared with a chi-square test. A P value of <0.05 was considered statistically significant.

## 3. Results

In total, 218 couples were included in this study. There were 42 (19.2%) couples in the endometriosis group, 116 (53.2%) couples in the unexplained infertility group, and 60 (27.6%) couples in the mild male infertility group. Basic demographic characteristics are presented in Table 1. The groups had similar demographic characteristics. The mean time interval between laparoscopic surgery and IUI was 2.1 ± 1.7 months in the endometriosis group. The mean TMSC of the mild male infertility group was 12.1 × 106, which was significantly lower than for the other groups (P < 0.001). 

**Table 1:** Demographic characteristics of subfertility groups.

	Endometriosis (n = 42)	Unexplained (n = 116)	Mild Male(n = 60)	P-value
Age, years	2 ± 9.1 ± 5.1	28.4 ± 4.3	29.2 ± 4.8	0.48
Duration of infertility, years	3.8 ± 1.9	3.6 ± 2.1	4.1 ± 2.5	0.35
Previous IUI, n (%)	2 (4.7)	7 (5.1)	4 (6.6)	0.92
Unilateral tubal blockage, n (%)	4 (9.5)	10 (8.6)	7 (11.6)	0.81
Mean antral follicle count	9.2 ± 1.1	9.1 ± 0.9	8.9 ± 0.7	0.20
Baseline FSH, IU/mL	7.9 ± 2.3	7.4 ± 3.1	7.7 ± 2.8	0.58
E2 (pg/mL)	40.2 ± 19.2	48.3 ± 18.3	46.8 ± 20.2	0.06
PRL (ng/mL)	18 ± 7.1	15.2 ± 7.2	17.2 ± 8.9	0.07
TSH (mIU/L)	2.1 ± 0.8	2.2 ± 0.9	1.9 ± 0.9	0.29
Total gonadotropin dose (IU)	970.4 ± 180.2	980.3 ± 170.3	930.4 ± 200.4	0.21
Duration of stimulation (days)	12.8 ± 3.9	11.0 ± 4.4	11.8 ± 4.3	0.60
TMSC (×106)	47.9 ± 7.2	44.3 ± 8.2	12.1 ± 4.1	<0.001

Note: The values are presented as mean. AFC: Atrial follicular count; TMSC: total motile sperm count.

The live birth rate of the entire study population was 9.6%. The live birth rates were 11.9%, 10.3%, and 6.6%, respectively, in the endometriosis, unexplained infertility, and mild male infertility groups (P = 0.63). In addition, there were no statistically significant differences between the groups for biochemical pregnancy and miscarriage rates (Table 2). 

**Table 2:** The pregnancy outcome among subfertility groups.

	Endometriosis group(n = 42)	Unexplained group(n = 116)	Mild male group(n = 60)	P
Biochemical pregnancy (%)	7 (16.6)	15 (12.9)	9 (15)	0.82
Live birth (%)	5 (11.9)	12 (10.3)	4 (6.6)	0.63
Miscarriage, 2(%)	2 (4.7)	3 (2.6)	5 (8.4)	0.22

## 4. Discussion 

In this study, we compared live birth rates between subfertile couples with mild male infertility, minimal–mild endometriosis, and unexplained infertility, and observed that live birth rates were similar between the groups. 

Controlled ovarian stimulation and IUI increases pregnancy rates in subfertile women regardless of infertility etiology; IUI without ovarian stimulation has no effect on live birth rates [18–22]. However, previous studies did not compare the pregnancy rates between different subfertility groups. A recent study from Brazil which included 237 IUI cycles in 198 patients concluded that infertility etiology did not affect pregnancy rates [23]. Although their results seem similar to ours, the primary outcome of that study was clinical pregnancy rate, not live birth rate. This was the greatest limitation of that study. 

Intrauterine insemination is a frequently used treatment option for subfertile couples. In our study, live birth rates were 10.3% and 11.9% in the unexplained infertility and endometriosis groups, respectively. Werbrouck et al. reported that pregnancy rates were similar between unexplained infertility and minimal–mild endometriosis groups (AFS stage I/II) within 6 months after surgical treatment [24]. Prado-Perez et al*.* also found no statistically significant differences in pregnancy rates of couples with unexplained infertility and stage I or II endometriosis who underwent IUI treatment [25]. The results of our study were in accordance with those of the aforementioned studies which showed similar live birth rates between unexplained infertility and minimal–mild endometriosis.

Although there was no statistically significant difference in live birth rate between different subfertility groups, it is important to note that the highest live birth rate was found in the endometriosis group (11.9%). Several studies have suggested lower pregnancy rates in couples with endometriosis than others following IUI [26,27]. However, we failed to demonstrate such a result. This might be as a result of the low number of subjects, as well as surgical treatment of endometriosis by laparoscopy. The benefits of laparoscopic surgery on pregnancy rates were also demonstrated in a previous Cochrane review [28]. In our study, complete laparoscopic surgical removal was performed in the endometriosis group before COS–IUI treatment, which could have increased the live birth rate. 

Miller et al. reported a 12.4% pregnancy rate per cycle when the TMSC was over 20 million, and 7.4% when the TMSC was between 10 and 20 million [29]. In our study, we found that the biochemical pregnancy rate was 15% and the live birth rate was 6.6% when mean TMSC was 11.6 million, which was in accordance with the abovementioned study. In the mild male infertility group, the miscarriage rate was the highest of all of the groups. We could not analyze any data other than motility with a spermiogram. The high miscarriage rate in the mild male infertility group could be related to sperm morphology, but we cannot comment further about the effect of the spermiogram because of the limitations of the retrospective study. On the other hand, a recent study which included 501 couples stated that abnormal sperm morphology did not impact live birth rates [30].

Success of IUI treatment is still a debate of importance for subfertile couples. The most recent NICE 2013 guidelines advised against offering routine IUI for people with unexplained infertility, mild endometriosis, or mild male factor infertility who are having regular unprotected sexual intercourse. According to the NICE guidelines, IVF should be considered after 2 years of unsuccessful conception National Institute for Health and Clinical Excellence (NICE) guidelines. 2013 Feb.https://www.nice.org.uk/guidance/cg156/ifp/chapter/intrauterine-insemination.. On the contrary, a recent review from 2017 suggested that IUI procedure should be undergone at least 3 cycles prior to in vitro fertilization (IVF) in couples with unexplained infertility and for men with a TMSC of >10 million [31]. No suggestion was presented for patients with mild endometriosis in that paper. According to the recent Cochrane review concerning male subfertility, there is no evidence of a difference in live birth rates between COS–IUI and timed intercourse [14]. They reported that this result was very low-quality evidence. On the other hand, we found similar pregnancy rates between the study groups. Although we did not compare live birth rates between subfertility etiology and timed intercourse, our results, especially in the male subfertility group, are valuable. We suggest COS–IUI treatment should be considered in couples with male subfertility before IVF procedures because of its substantial live birth rate, its simplicity, and low cost.

The main strengths of the present study were using live birth rate as the primary outcome measure and evaluating only the first COS–IUI cycle of each couple to prevent crossover bias. The major limitations of our study were the retrospective design and the low number of subjects, particularly in the endometriosis group. However, we could not include more subjects in such a study using strict inclusion and exclusion criteria conducted in a single center. Another limitation of the study was the lack of a hypothetical power analysis. 

In conclusion, different subfertility etiologies do not affect the success of COS–IUI treatment in terms of live birth rate. The success rate of mild male subfertility following a COS–IUI cycle for live birth rates is similar to those of the endometriosis and unexplained subfertility groups. Further large prospective studies are needed to determine the exact effect of subfertility etiology on the success of COS–IUI treatment.
